# Supplementary material for: DNA Barcoding and Fertilization Strategies in Sideritis syriaca subsp. syriaca, a Local Endemic Plant of Crete with High Medicinal Value
Source: Int J Mol Sci. 2024 Feb 4;25(3):1891. doi: 10.3390/ijms25031891 (PMC10856587; doi:10.3390/ijms25031891)
Supplement: Supplementary file 1 [file ijms-25-01891-s001.zip › ijms-2799551-supplementary.pdf]

Supplementary material Methods S1. Chemical composition of fertilizers used in the experimental procedure (Theofrastos company, Korinthos, Greece) and fertilization treatments applied in the pilot cultivation of *Sideritis syriaca* subsp. *syriaca* with other Cretan endemic plants at the premises of the Hellenic Mediterranean University

Scheme A. Integrated nutrient management (INM) by foliar application (INM-fa): The nutrient solution consisted of THEORUN at 7 ml L^{-1}, THEOCAL at 1.5 g L^{-1}, THEOFAST at 5 ml L^{-1}, 10-47-10 (AGRI.FE.M. LTD Fertilizers, Greece) at 3.2 g L^{-1}, K_2SO_4 (0-0-52, AGRI.FE.M. LTD Fertilizers, Greece) at 2.07 g L^{-1}, micronutrients (Plex Mix, AGRI.FE.M. LTD Fertilizers, Greece) at 1.5 ml L^{-1} and $MgSO_4$ (Mg 25.6%, AGRI.FE.M. LTD Fertilizers, Greece) at 0.6 g L^{-1}.

Scheme B. Conventional inorganic fertilization by foliar application (ChF-fa): The nutrient solution consisted of NH_4NO_3 (34,4-0-0, Neofert®, Neochim PLC, Bulgaria) at 2.7 g L^{-1}, $Ca(NO_3)_2$ (NITROCAL, Agrohimiki, Greece) at 1.7 g L^{-1}, 10-47-10 at 3.2 g L^{-1}, K_2SO_4 (0-0-52) at 2.27 g L^{-1}, micronutrients Plex Mix at 1.5 ml L^{-1} and $MgSO_4$ (Mg 25.6 %) at 0.6 g L^{-1}.

Scheme C. Control, with foliar and soil applications with tap water.

Scheme D. INM by soil application (INM-sa): The nutrient solution consisted of THEORUN at 7 ml L^{-1}, THEOCAL at 1.5 g L^{-1}, THEOMASS at 10 ml L^{-1}, 10-47-10 at 3.2 g L^{-1}, K_2SO_4 (0-0-52) at 2.1 g L^{-1}, micronutrients Plex Mix at 1.5 ml L^{-1} and $MgSO_4$ (Mg 25.6 %) at 0.3 g L^{-1}.

Scheme E. Conventional inorganic fertilization by soil application (ChF-sa): The nutrient solution was consisted of NH_4NO_3 (34,4-0-0) at 2.7 g L^{-1}, $Ca(NO_3)_2$ (NITROCAL) at 1.7 g L^{-1}, 10-47-10 at 3.2 g L^{-1}, K_2SO_4 (0-0-52) at 2.3 g L^{-1}, micronutrients, Plex Mix at 1.5 ml L^{-1} and $MgSO_4$ (Mg 25.6 %) at 0.3 g L^{-1}.

Scheme F. Mixture of plant extracts as biostimulant by soil application (MPE-sa): The nutrient solution consisted of THEOMASS at 10 ml L^{-1}.

Supplementary Material Table S1. Alignment presenting the nucleotide differences among 15 *Sideritis* species based on the molecular plastid markers *rbc*L and *trn*L/*trn*F. Accession numbers of DNA sequences obtained in this study from the GenBank are indicated next to taxon names. The clade marked in orange represents *S. syriaca* subsp. *syriaca* GR-1-BBGK-15,5939 studied herein. The taxon marked with asterisk (*) is characterized as *S. syriaca* in the database, but based on the origin of the specimen, it should be identified as *S. euboea* due to the narrow distribution of *S. syriaca* in Crete.

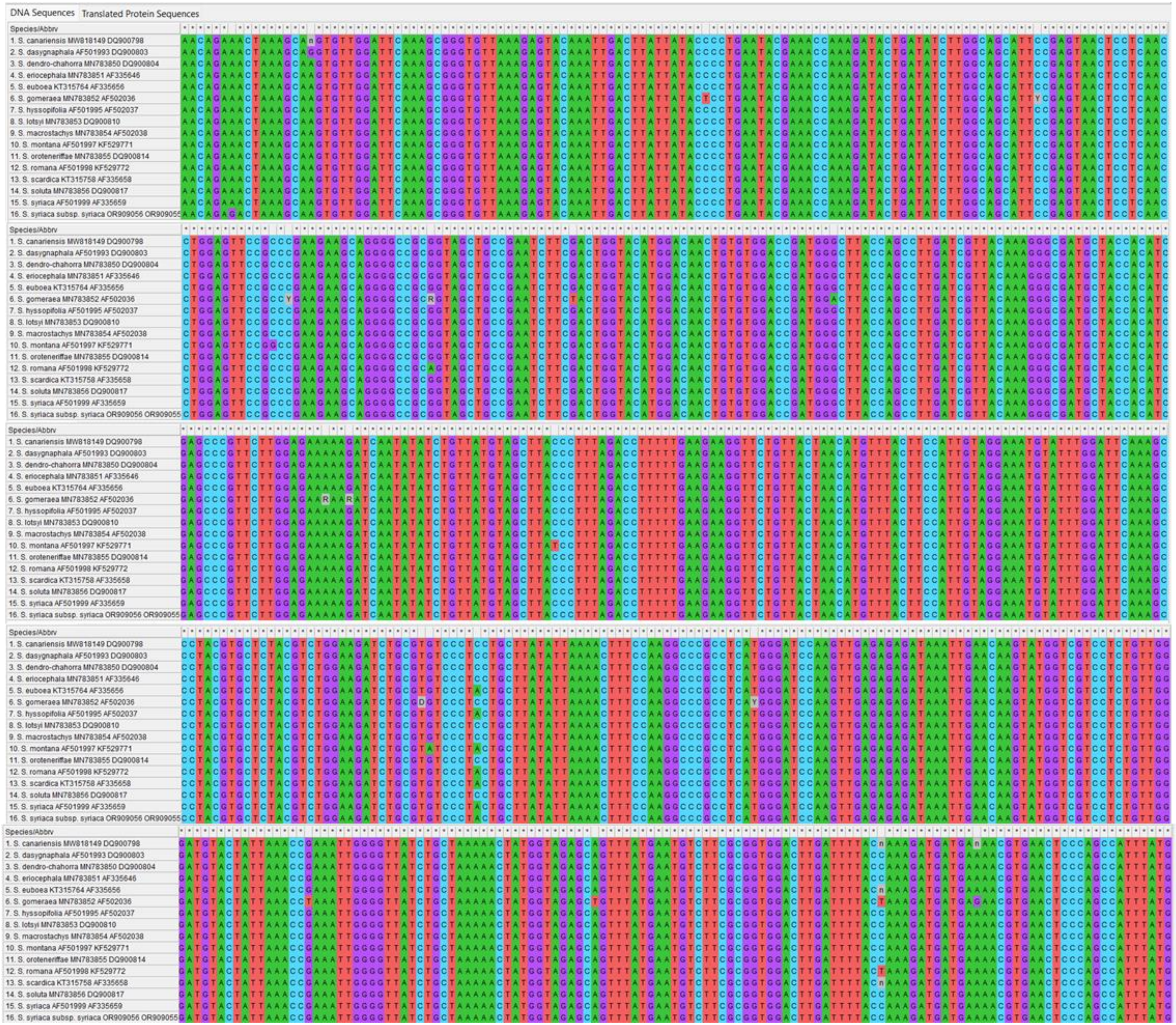
DNA Sequences
Translated Protein Sequences
Species/Abbrv
1. S. canariensis MW818149 DQ900798
2. S. dasygnaphala AF501993 DQ900803
3. S. dendro-chahorra MN783850 DQ900804
4. S. eriocephala MN783851 AF335646
5. S. euboea KT315764 AF335656
6. S. gomeraea MN783852 AF502036
7. S. hyssopifolia AF501995 AF502037
8. S. lotsyi MN783853 DQ900810
9. S. macrostachys MN783854 AF502038
10. S. montana AF501997 KF529771
11. S. oroteneriffae MN783855 DQ900814
12. S. romana AF501998 KF529772
13. S. scardica KT315758 AF335658
14. S. soluta MN783856 DQ900817
15. S. syriaca AF501999 AF335659
16. S. syriaca subsp. syriaca OR909056 OR909055

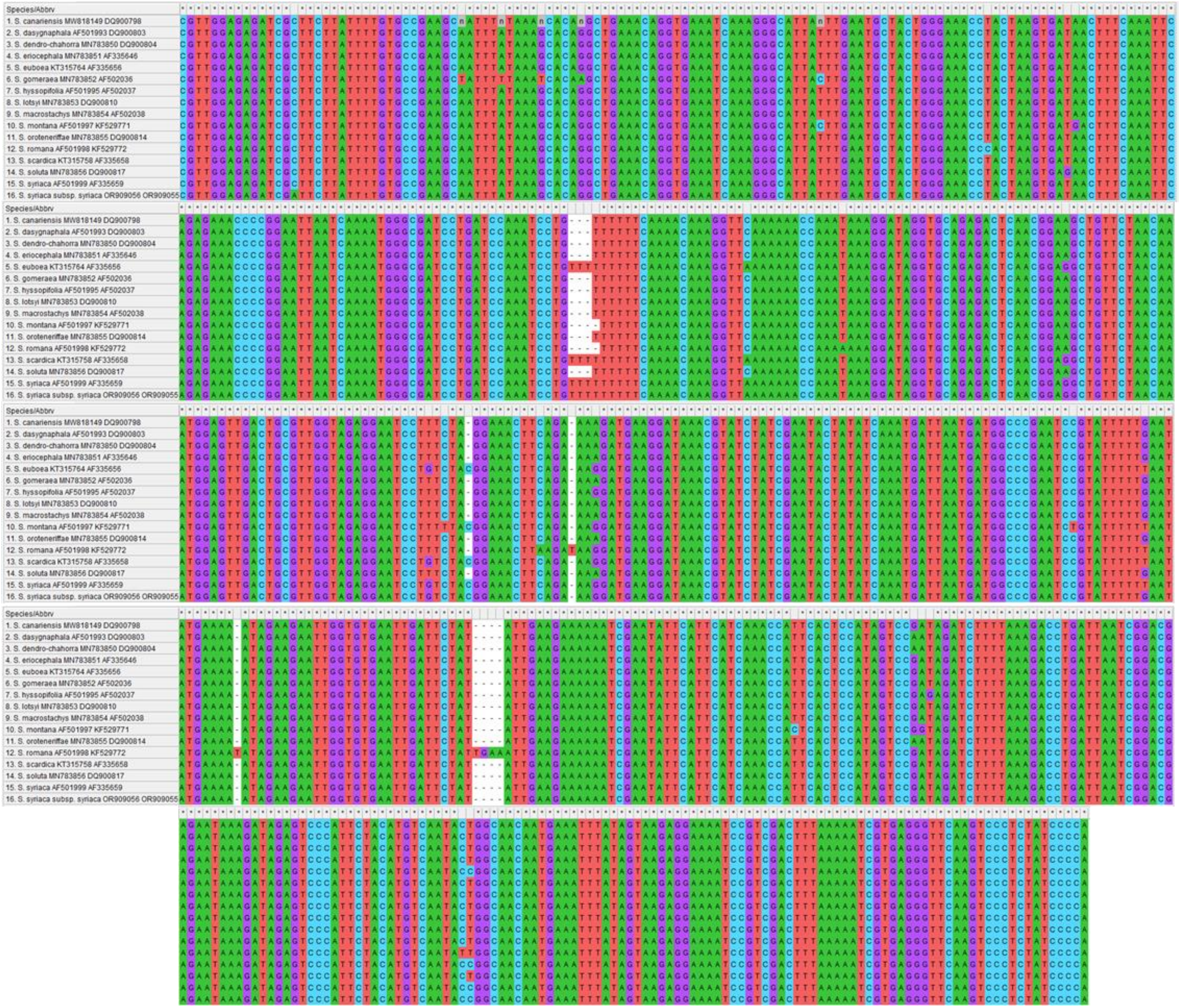

Species/Abbrv
1. S. canariensis MW818149 DQ900798
2. S. dasygnaphala AF501993 DQ900803
3. S. dendro-chahorra MN783850 DQ900804
4. S. eriocephala MN783851 AF335646
5. S. euboea KT315764 AF335656
6. S. gomeraea MN783852 AF502036
7. S. hyssopifolia AF501995 AF502037
8. S. lotsyi MN783853 DQ900810
9. S. macrostachys MN783854 AF502038
10. S. montana AF501997 KF529771
11. S. oroteneriffae MN783855 DQ900814
12. S. romana AF501998 KF529772
13. S. scardica KT315758 AF335658
14. S. soluta MN783856 DQ900817
15. S. syriaca AF501999 AF335659
16. S. syriaca subsp. syriaca OR909056 OR909055

Supplementary Material Table S2 Phylogenetic analysis of *Sideritis* taxa using the plastid molecular marker *rbc*L. Alignment of 20 *Sideritis* DNA sequences retrieved from GenBank compared to *Sideritis syriaca* subsp. *syriaca* GR-1-BBGK-15,5939 specimen of the current study (OP909056); the conserved areas (100%) are toggled.

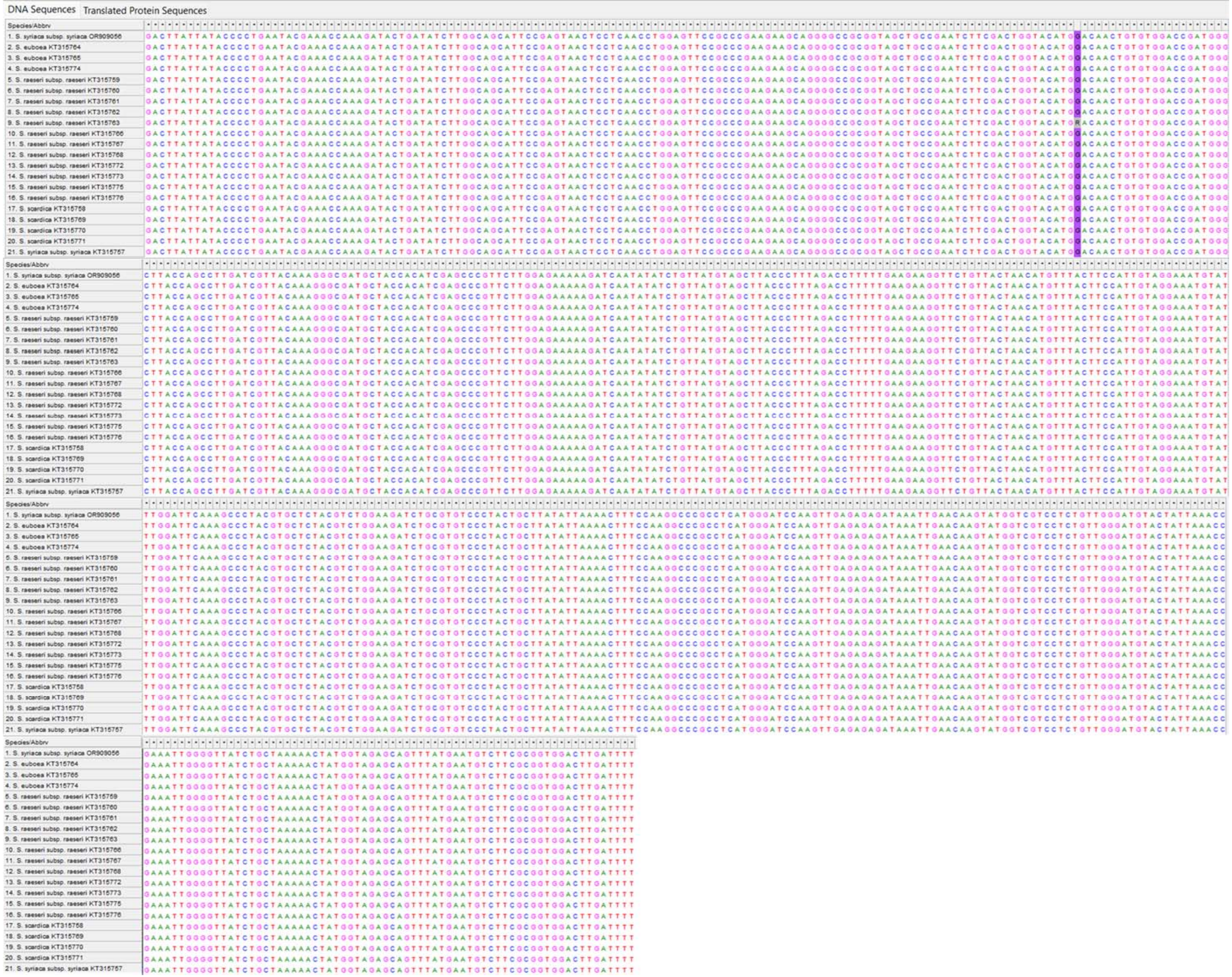
DNA Sequences
Translated Protein Sequences
Species/Abbrv
1. S. syriaca subsp. syriaca OR909056
2. S. euboea KT315764
3. S. euboea KT315765
4. S. euboea KT315774
5. S. raeseri subsp. raeseri KT315759
6. S. raeseri subsp. raeseri KT315760
7. S. raeseri subsp. raeseri KT315761
8. S. raeseri subsp. raeseri KT315762
9. S. raeseri subsp. raeseri KT315763
10. S. raeseri subsp. raeseri KT315766
11. S. raeseri subsp. raeseri KT315767
12. S. raeseri subsp. raeseri KT315768
13. S. raeseri subsp. raeseri KT315772
14. S. raeseri subsp. raeseri KT315773
15. S. raeseri subsp. raeseri KT315775
16. S. raeseri subsp. raeseri KT315776
17. S. scardica KT315758
18. S. scardica KT315769
19. S. scardica KT315770
20. S. scardica KT315771
21. S. syriaca subsp. syriaca KT315757

Supplementary Material Table S3 Phylogenetic analysis of *Sideritis* taxa using the plastid molecular marker *psb*A-*trn*H. Alignment of 94 *Sideritis* DNA sequences retrieved from GenBank compared to *Sideritis syriaca* subsp. *syriaca* GR-1-BBGK-15,5939 specimen of the current study (OP909054); the conserved areas (100%) are toggled.

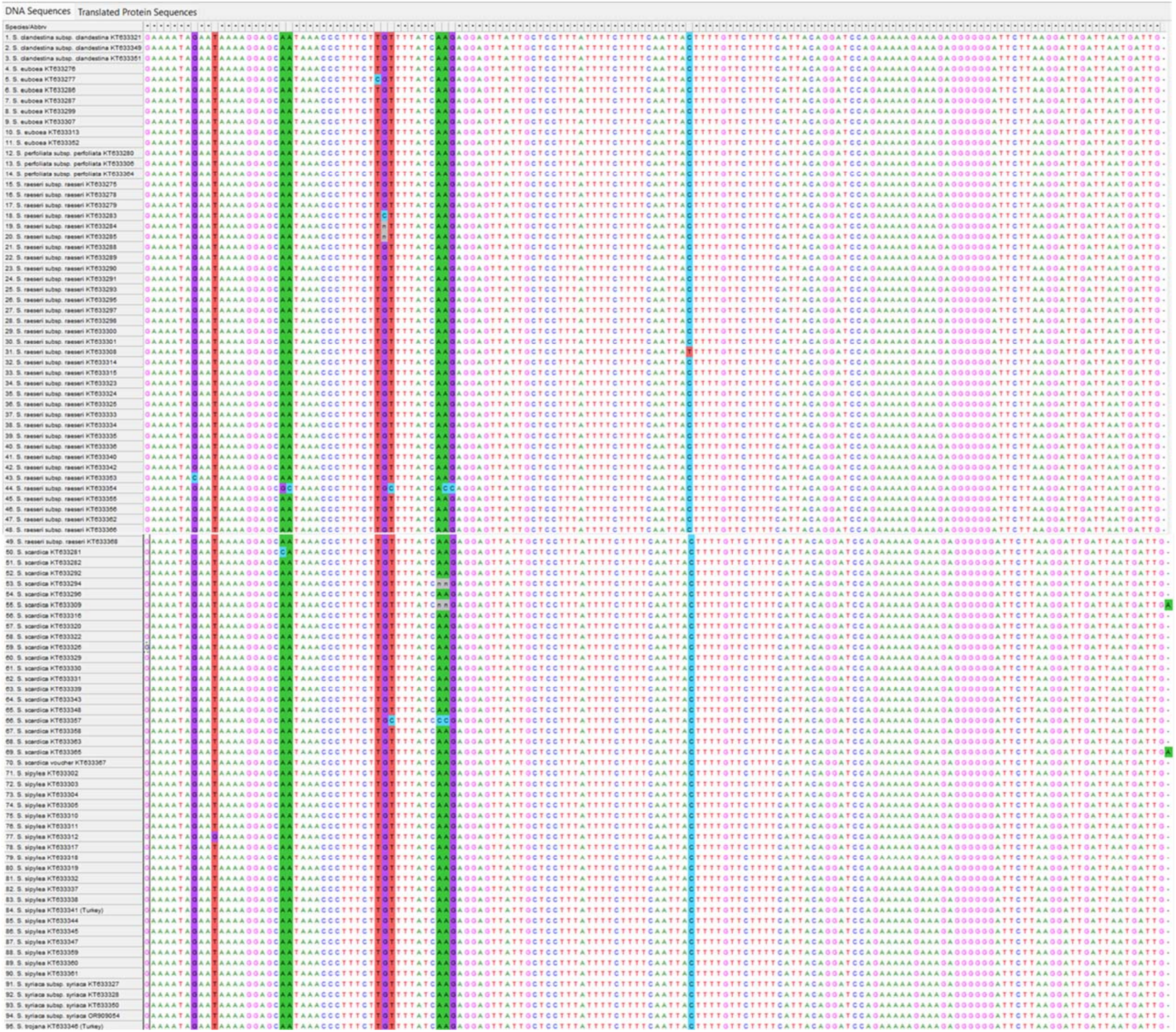
DNA Sequences
Translated Protein Sequences

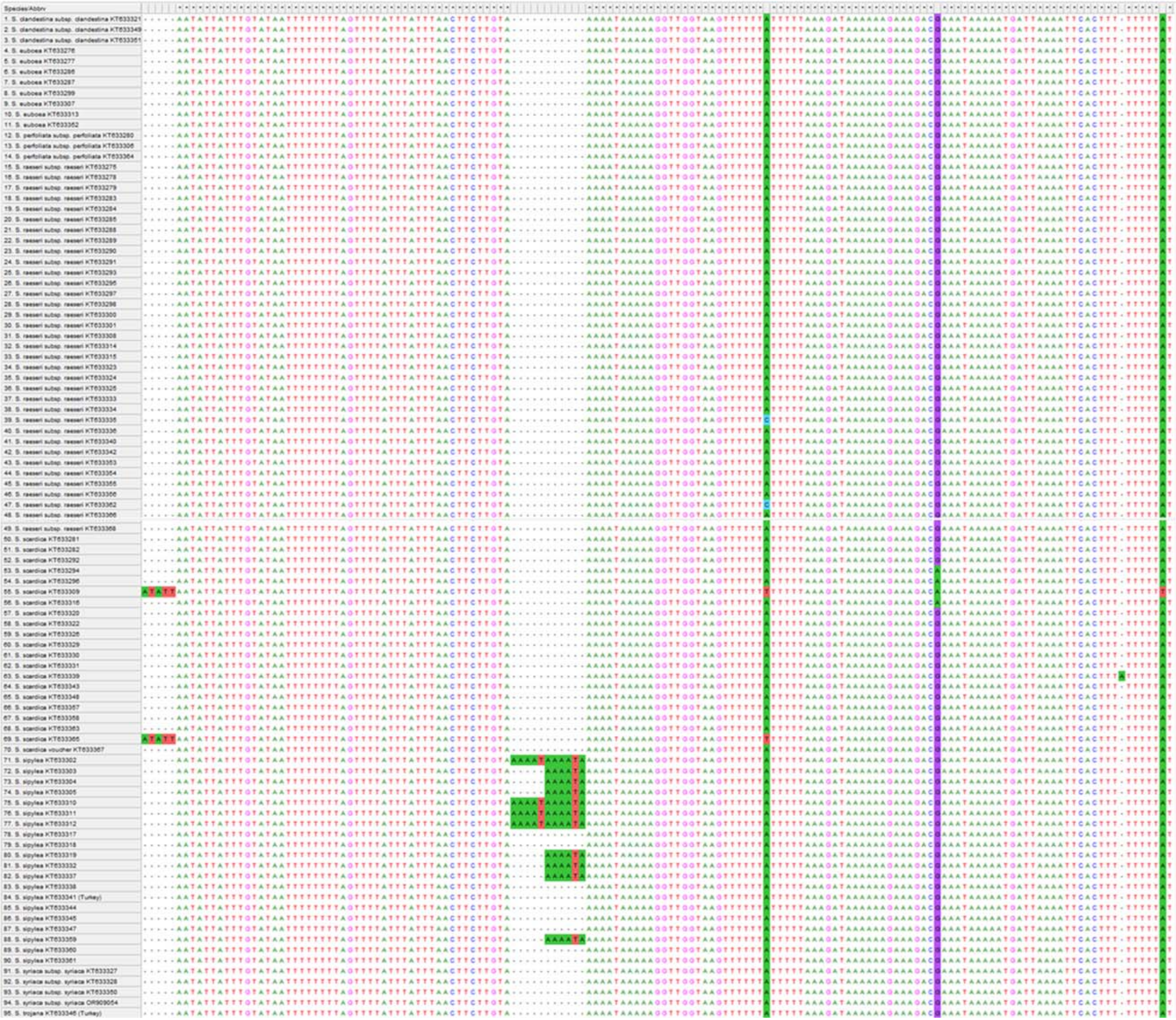

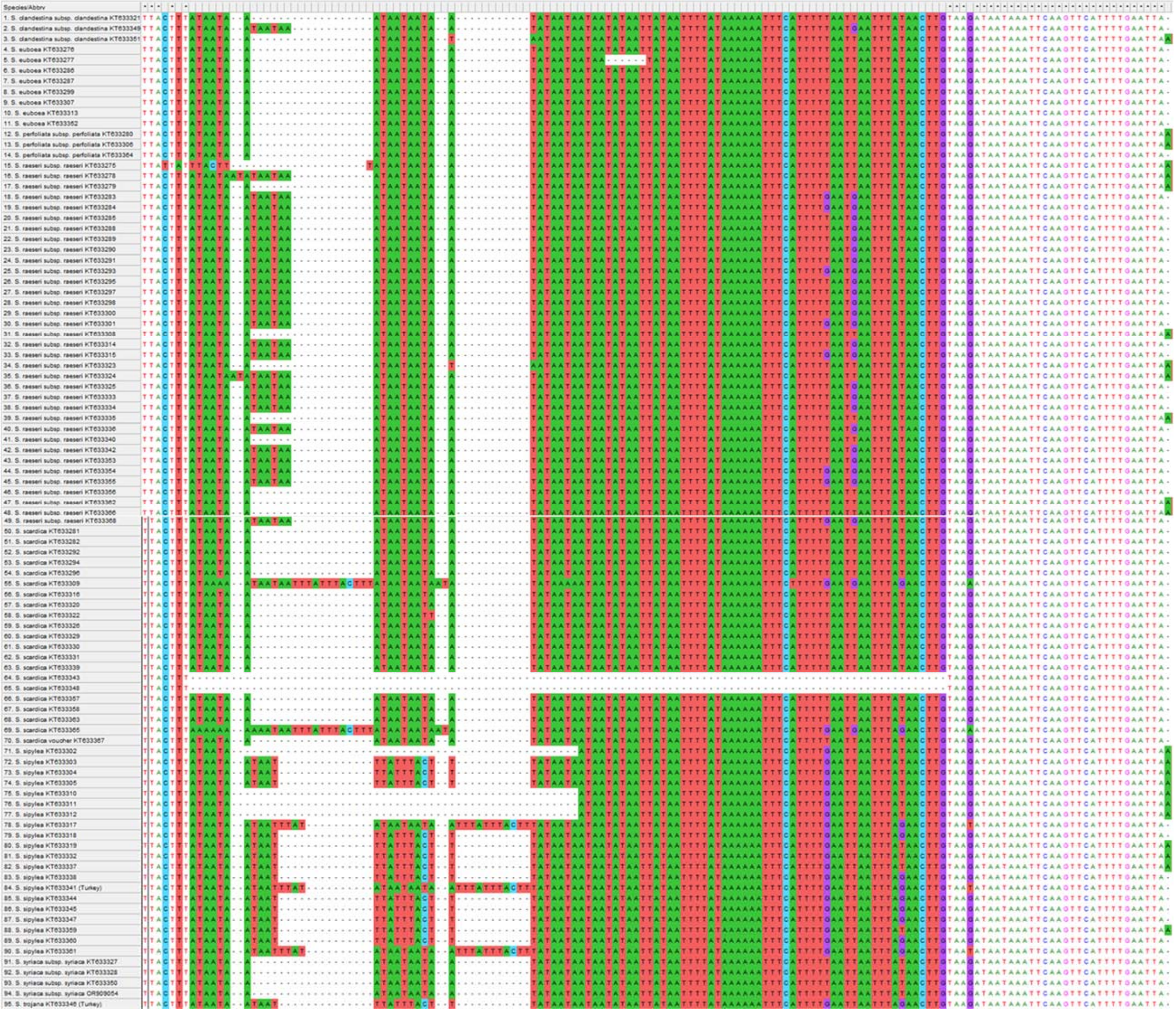
Species/Abbrv
1. S. clandestina subsp. clandestina KT633321
2. S. clandestina subsp. clandestina KT633349
3. S. clandestina subsp. clandestina KT633351
4. S. euboea KT633276
5. S. euboea KT633277
6. S. euboea KT633286
7. S. euboea KT633287
8. S. euboea KT633299
9. S. euboea KT633307
10. S. euboea KT633313
11. S. euboea KT633352
12. S. perfoliata subsp. perfoliata KT633280
13. S. perfoliata subsp. perfoliata KT633306
14. S. perfoliata subsp. perfoliata KT633364
15. S. raeseri subsp. raeseri KT633275
16. S. raeseri subsp. raeseri KT633278
17. S. raeseri subsp. raeseri KT633279
18. S. raeseri subsp. raeseri KT633283
19. S. raeseri subsp. raeseri KT633284
20. S. raeseri subsp. raeseri KT633285
21. S. raeseri subsp. raeseri KT633288
22. S. raeseri subsp. raeseri KT633289
23. S. raeseri subsp. raeseri KT633290
24. S. raeseri subsp. raeseri KT633291
25. S. raeseri subsp. raeseri KT633293
26. S. raeseri subsp. raeseri KT633295
27. S. raeseri subsp. raeseri KT633297
28. S. raeseri subsp. raeseri KT633298
29. S. raeseri subsp. raeseri KT633300
30. S. raeseri subsp. raeseri KT633301
31. S. raeseri subsp. raeseri KT633308
32. S. raeseri subsp. raeseri KT633314
33. S. raeseri subsp. raeseri KT633315
34. S. raeseri subsp. raeseri KT633323
35. S. raeseri subsp. raeseri KT633324
36. S. raeseri subsp. raeseri KT633325
37. S. raeseri subsp. raeseri KT633333
38. S. raeseri subsp. raeseri KT633334
39. S. raeseri subsp. raeseri KT633335
40. S. raeseri subsp. raeseri KT633336
41. S. raeseri subsp. raeseri KT633340
42. S. raeseri subsp. raeseri KT633342
43. S. raeseri subsp. raeseri KT633353
44. S. raeseri subsp. raeseri KT633354
45. S. raeseri subsp. raeseri KT633355
46. S. raeseri subsp. raeseri KT633356
47. S. raeseri subsp. raeseri KT633362
48. S. raeseri subsp. raeseri KT633366
49. S. raeseri subsp. raeseri KT633368
50. S. scardica KT633281
51. S. scardica KT633282
52. S. scardica KT633292
53. S. scardica KT633294
54. S. scardica KT633296
55. S. scardica KT633309
56. S. scardica KT633316
57. S. scardica KT633320
58. S. scardica KT633322
59. S. scardica KT633326
60. S. scardica KT633329
61. S. scardica KT633330
62. S. scardica KT633331
63. S. scardica KT633339
64. S. scardica KT633343
65. S. scardica KT633348
66. S. scardica KT633357
67. S. scardica KT633358
68. S. scardica KT633363
69. S. scardica KT633365
70. S. scardica voucher KT633367
71. S. sipylea KT633302
72. S. sipylea KT633303
73. S. sipylea KT633304
74. S. sipylea KT633305
75. S. sipylea KT633310
76. S. sipylea KT633311
77. S. sipylea KT633312
78. S. sipylea KT633317
79. S. sipylea KT633318
80. S. sipylea KT633319
81. S. sipylea KT633332
82. S. sipylea KT633337
83. S. sipylea KT633338
84. S. sipylea KT633341 (Turkey)
85. S. sipylea KT633344
86. S. sipylea KT633345
87. S. sipylea KT633347
88. S. sipylea KT633359
89. S. sipylea KT633360
90. S. sipylea KT633361
91. S. syriaca subsp. syriaca KT633327
92. S. syriaca subsp. syriaca KT633328
93. S. syriaca subsp. syriaca KT633350
94. S. syriaca subsp. syriaca OR909054
95. S. trojana KT633346 (Turkey)

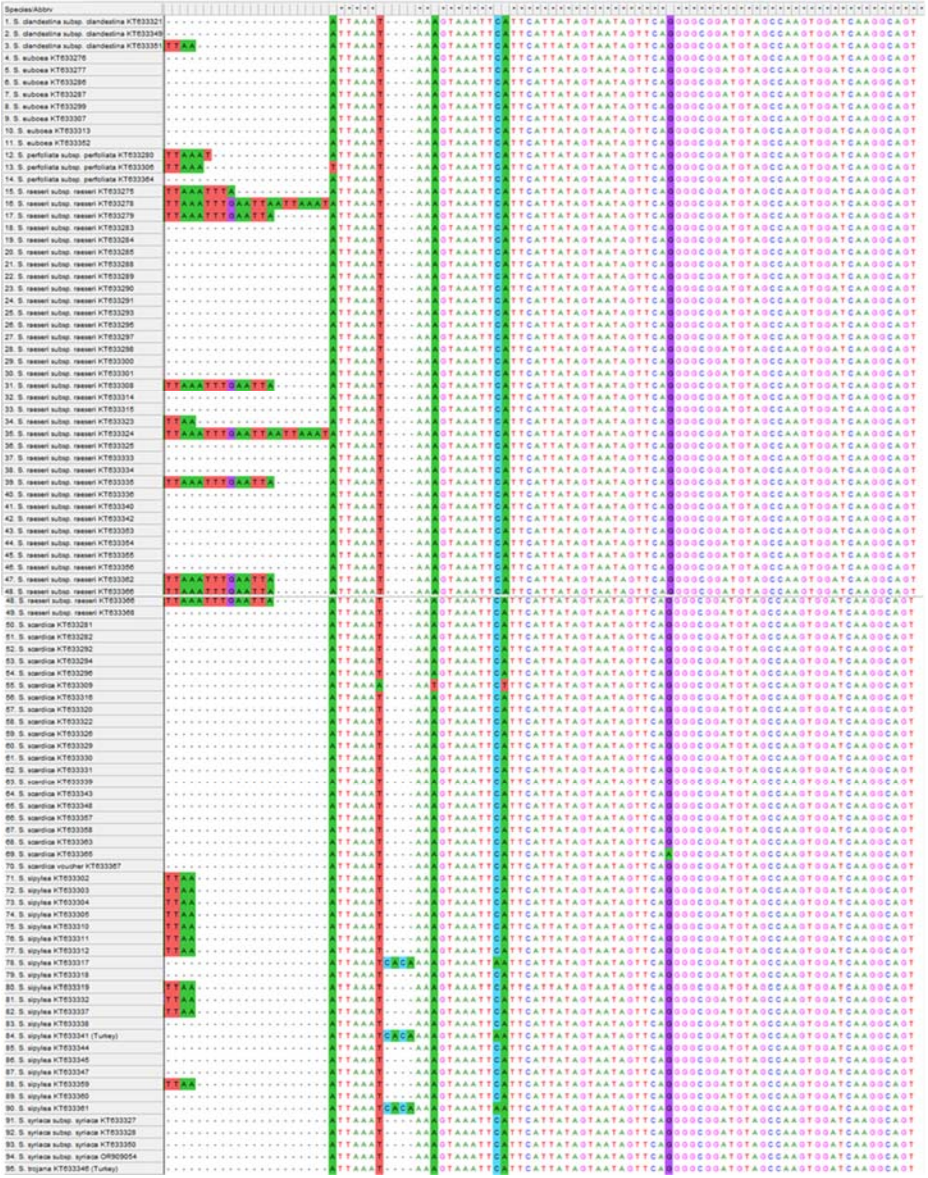

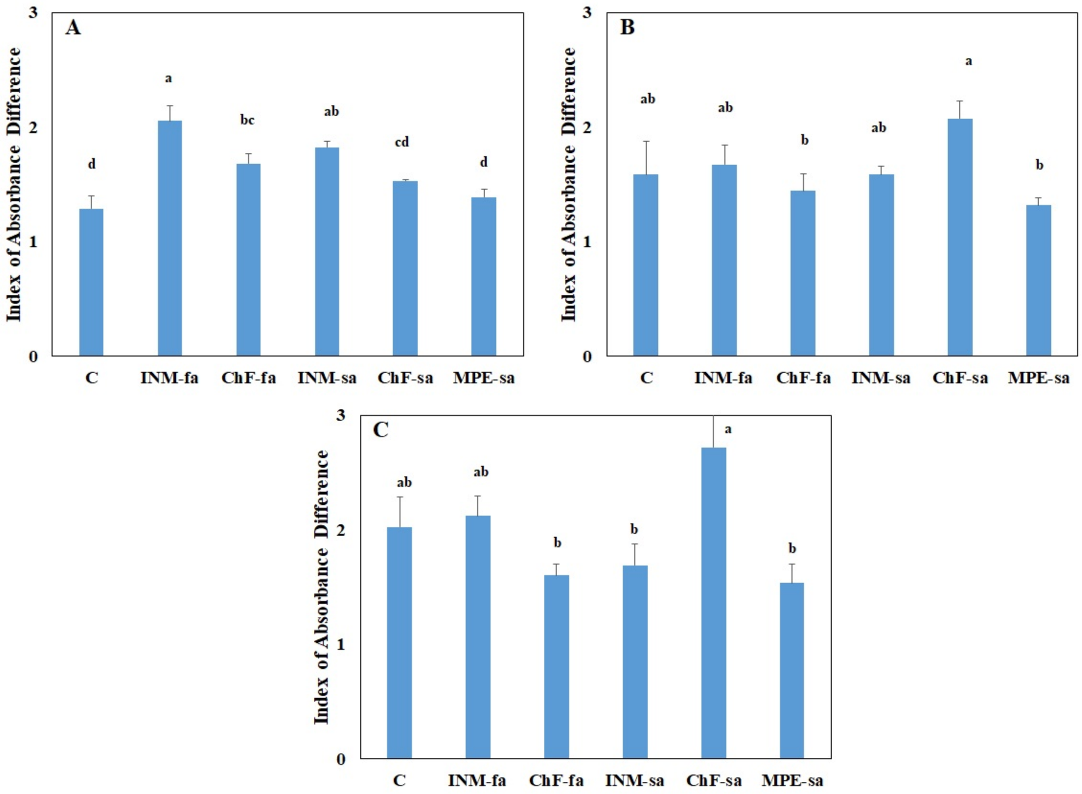

Supplementary Figure S1. Effect of fertilization scheme applied through foliar or soil on leaf index of absorbance difference of *Sideritis syriaca* subsp. *syriaca* at vegetative (A), early flowering (B), and full flowering (C) stage. C: Control (water); INM-fa: Integrated nutrient management (INM) by foliar application; ChF-fa: Conventional inorganic fertilization (ChF) by foliar application; INM-sa: INM by soil application; ChF-sa: ChF by soil application; MPE-sa: Mixture of plant extracts as biostimulant by soil application (THEOMASS). Columns represent the mean of three replicates ± SEM. Within each plot, different letters indicate significant differences among means.

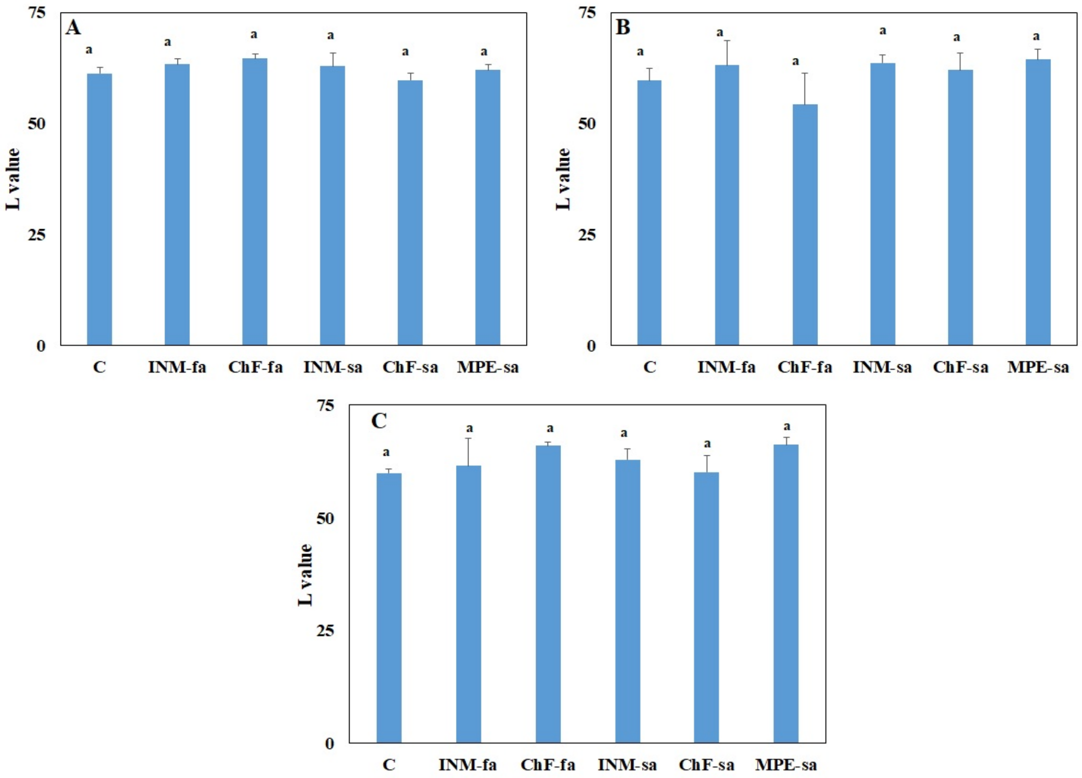

Supplementary Figure S2. Effect of fertilization scheme applied through foliar or soil on leaf L value of *Sideritis syriaca* subsp. *syriaca* at vegetative (A), early flowering (B), and full flowering (C) stage. C: Control (water); INM-fa: Integrated nutrient management (INM) by foliar application; ChF-fa: Conventional inorganic (ChF) fertilization by foliar application; INM-sa: INM by soil application; ChF-sa: ChF by soil application; MPE-sa: Mixture of plant extracts as biostimulant by soil application (THEOMASS). Columns represent the mean of three replicates ± SEM. Within each plot, different letters indicate significant differences among means.

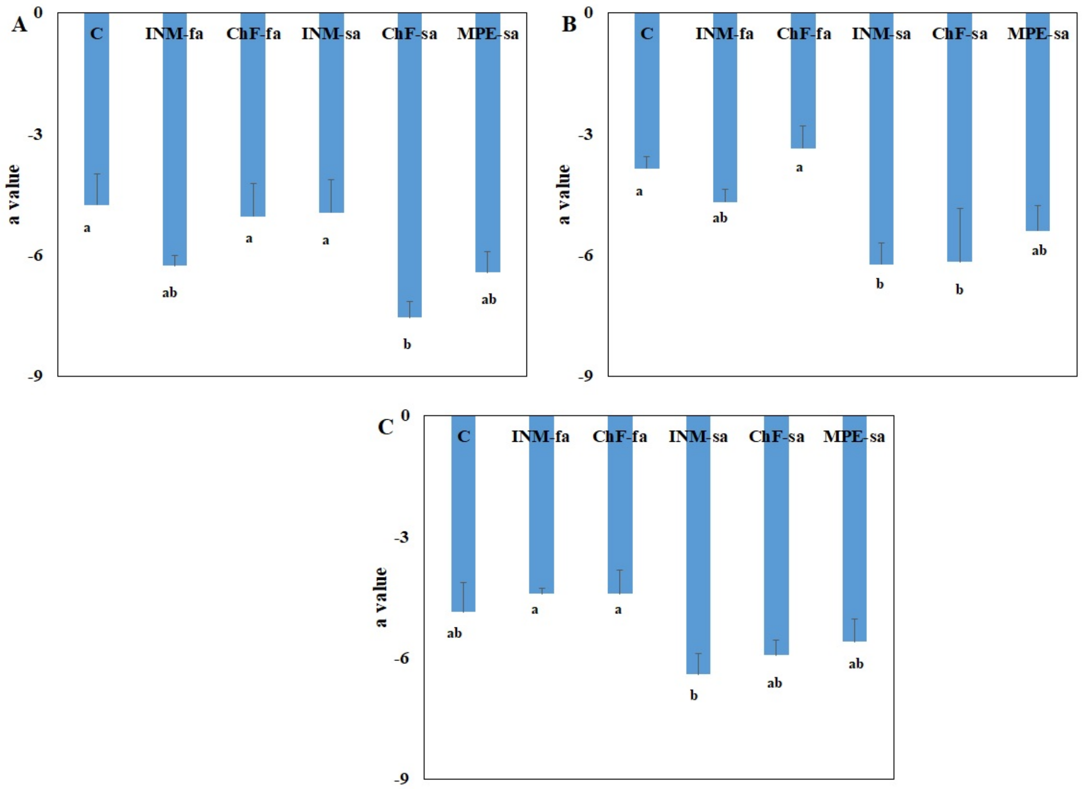

Supplementary Figure S3. Effect of fertilization scheme applied through foliar or soil on leaf a value of *Sideritis syriaca* subsp. *syriaca* at vegetative (A), early flowering (B), and full flowering (C) stage. C: Control (water); INM-fa: Integrated nutrient management (INM) by foliar application; ChF-fa: Conventional inorganic fertilization (ChF) by foliar application; INM-sa: INM by soil application; ChF-sa: ChF by soil application; MPE-sa: Mixture of plant extracts as biostimulant by soil application (THEOMASS). Columns represent the mean of three replicates ± SEM. Within each plot, different letters indicate significant differences among means.

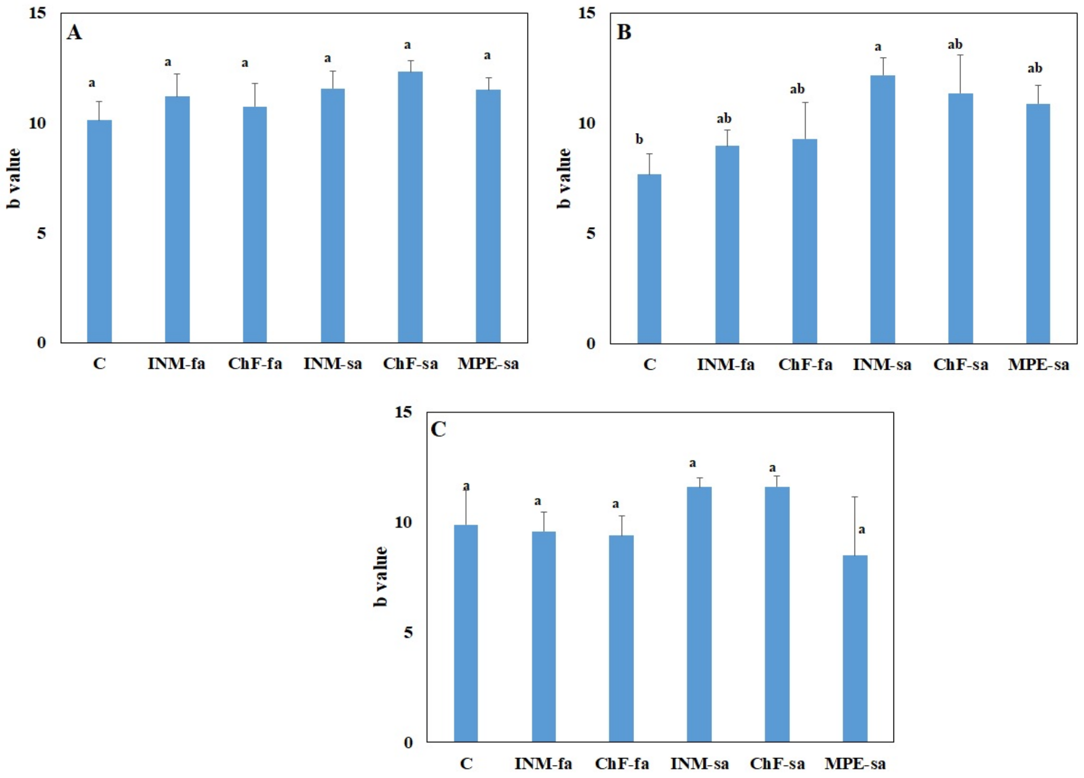

Supplementary Figure S4. Effect of fertilization scheme applied through foliar or soil on leaf b value of *Sideritis syriaca* subsp. *syriaca* at vegetative (A), early flowering (B), and full flowering (C) stage. C: Control (water); INM-fa: Integrated nutrient management (INM) by foliar application; ChF-fa: Conventional inorganic fertilization (ChF) by foliar application; INM-sa: INM by soil application; ChF-sa: ChF by soil application; MPE-sa: Mixture of plant extracts as biostimulant by soil application (THEOMASS). Columns represent the mean of three replicates ± SEM. Within each plot, different letters indicate significant differences among means.
